# Multigene mutational profiling of cholangiocarcinomas identifies actionable molecular subgroups

**DOI:** 10.18632/oncotarget.1943

**Published:** 2014-05-01

**Authors:** Michele Simbolo, Matteo Fassan, Andrea Ruzzenente, Andrea Mafficini, Laura D. Wood, Vincenzo Corbo, Davide Melisi, Giuseppe Malleo, Caterina Vicentini, Giorgio Malpeli, Davide Antonello, Nicola Sperandio, Paola Capelli, Anna Tomezzoli, Calogero Iacono, Rita T. Lawlor, Claudio Bassi, Ralph H. Hruban, Alfredo Guglielmi, Giampaolo Tortora, Filippo de Braud, Aldo Scarpa

**Affiliations:** ^1^ ARC-Net Research Centre, University and Hospital Trust of Verona, Verona, Italy; ^2^ Department of Pathology and Diagnostics, University and Hospital Trust of Verona, Verona, Italy; ^3^ Department of Surgery, General Surgery A, University of Verona, Verona, Italy; ^4^ Department of Pathology, Johns Hopkins University and Sidney Kimmel Comprehensive Cancer Center, Baltimore, MD, USA; ^5^ Department of Medical Oncology, University and Hospital Trust of Verona, Verona, Italy; ^6^ Department of Surgery, General Surgery B, University of Verona, Verona, Italy; ^7^ Medical Oncology Unit 1, Fondazione Istituto di Ricovero e Cura a Carattere Scientifico, Istituto Nazionale dei Tumori, Milan, Italy

**Keywords:** cholangiocarcinoma, next-generation sequencing, molecular subclassification, target therapy, multigene mutational panels

## Abstract

One-hundred-fifty-three biliary cancers, including 70 intrahepatic cholangiocarcinomas (ICC), 57 extrahepatic cholangiocarcinomas (ECC) and 26 gallbladder carcinomas (GBC) were assessed for mutations in 56 genes using multigene next-generation sequencing. Expression of EGFR and mTOR pathway genes was investigated by immunohistochemistry. At least one mutated gene was observed in 118/153 (77%) cancers. The genes most frequently involved were *KRAS* (28%), *TP53* (18%), *ARID1A* (12%), *IDH1/2* (9%), *PBRM1* (9%), *BAP1* (7%), and *PIK3CA* (7%). *IDH1/2* (p=0.0005) and *BAP1* (p=0.0097) mutations were characteristic of ICC, while *KRAS* (p=0.0019) and *TP53* (p=0.0019) were more frequent in ECC and GBC. Multivariate analysis identified tumour stage and *TP53* mutations as independent predictors of survival. Alterations in chromatin remodeling genes (*ARID1A*, *BAP1*, *PBRM1*, *SMARCB1*) were seen in 31% of cases. Potentially actionable mutations were seen in 104/153 (68%) cancers: i) *KRAS/NRAS/BRAF* mutations were found in 34% of cancers; ii) mTOR pathway activation was documented by immunohistochemistry in 51% of cases and by mutations in mTOR pathway genes in 19% of cancers; iii) TGF-ß/Smad signaling was altered in 10.5% cancers; iv) mutations in tyrosine kinase receptors were found in 9% cases. Our study identified molecular subgroups of cholangiocarcinomas that can be explored for specific drug targeting in clinical trials.

## INTRODUCTION

Cholangiocarcinoma is a phenotypical and clinical heterogeneous collection of biliary tract malignancies, classified according to the World Health Organization (WHO) as intrahepatic (ICC) or extrahepatic cholangiocarcinomas (ECC) [1, 2]. The former arise in the substance of the liver, the latter in large extrahepatic ducts, i.e. hepatic ducts and common bile duct. Gallbladder carcinomas (GBC) also have biliary epithelial differentiation. Clinically, both cholangiocarcinomas and GBC have very poor prognosis. Surgical resection is the only potentially curative therapy, but most cases are inoperable [3-7]. In contrast to other solid tumours, no effective molecular targeted agent has been approved for biliary tract cancers, and patients have limited access to clinical trials [8-11].

Previous studies on molecular alterations in biliary tract cancers have focused on selected genes, including those altered in pancreatic adenocarcinoma (*KRAS*, *TP53*, *CDKN2A* and *SMAD4*) [12]. Mutations in *PIK3CA*, *PTEN*, *AKT1*, *IDH1* and *IDH2* have been reported in this class of tumours [13-20]. However, the prevalence of these alterations varies widely among studies. Two recent whole exome-sequencing studies of ICC revealed a key role for chromatin remodeling genes *BAP1*, *ARID1A* and *PBRM1* in the development of these tumours [13, 21].

The validation of whole exome studies by sequencing analysis of hotspot mutations in larger and characterized series has been a fruitful approach in identifying potential targets for personalized therapy for several malignancies [22]. Next-generation sequencing (NGS) has been recently introduced and is the most sensitive approach to simultaneously characterize multiple genes starting from a limited amount of DNA, also DNA derived from formalin-fixed paraffin-embedded (FFPE) samples [13, 23-25].

In the present study, we assayed the mutational status of 56 cancer-related genes in 153 biliary tract cancers, using a targeted next-generation sequencing methodology, with the aim of identifying molecular subgroups driving the development of personalized therapy approaches for patients affected by these neoplasms.

## RESULTS

### Clinico-pathological characteristics of the series

Patients' demographic and clinico-pathological data are summarized in Table 1. Mean tumour size was 4.8±3.4 cm (median=6.5; range=0.5-20.0), and was significantly higher in ICC than ECC and GBC (*p*=9.77 E^−12^). Synchronous multinodular lesions were found in 31 cases (20.3%). Tumour grading was G1 in 20, G2 in 94, and G3 in 38 cases, while the remaining case was undifferentiated.

**Table 1:** Clinico-pathological features of 153 biliary carcinomas

		Total (n=153)	ICC (n=70)	ECC (n=57)	GBC (n=26)	P-value*
Sex		59F 94M	28F 42M	20F 37M	11F 15M	0.776
Age		65.4±10.8	64.8±11.6	64.2±10.8	69.6±8.0	0.118
Dimension (cm)		4.8±3.4	6.6±3.7	2.6±1.3	3.3±1.4	9.77 E-12
Multiple nodes		31 (20.3%)	28 (40.0%)	3 (5.3%)	-	2.84 E-6
Grade	1	20 (13.1%)	7 (10.0%)	8 (14.0%)	5 (19.2%)	0.630
	2	94 (61.4%)	45 (64.3%)	36 (63.2%)	13 (50.0%)	
	3	38 (24.8%)	18 (25.7%)	12 (21.0%)	8 (30.7%)	
	4	1 (0.6%)	0 (0.0%)	1 (1.8%)	0 (0.0%)	
Presence of BiIIN		49 (32.0%)	11 (15.7%)	24 (42.1%)	14 (53.8%)	0.0002
Vascular invasion		107 (70.0%)	51 (72.9%)	38 (66.7%)	18 (69.2%)	0.773
Perineural invasion		93 (60.8%)	31 (44.3%)	44 (77.2%)	18 (69.2%)	0.0005
Radicality of resection	R0	110 (71.9%)	56 (80.0%)	33 (57.9%)	19 (73.1%)	0.068
	R1	43 (28.1%)	14 (20.0%)	24 (42.1%)	7 (26.9%)	
HBV/HCV infection		22 (14.4%)	17 (24.3%)	3 (5.3%)	2 (7.7%)	0.041
Cirrhosis		13 (8.5%)	10 (14.3%)	3 (5.3%)	0 (0.0%)	0.005
Stage	I	20 (13.1%)	12 (17.1%)	5 (8.8%)	3 (11.5%)	0.034
	II	51 (33.3%)	21 (30.0%)	22 (38.6%)	8 (30.7%)	
	III	43 (28.1%)	12 (17.1%)	20 (35.1%)	11 (42.3%)	
	IV	39 (25.5%)	25 (35.8%)	10 (17.5%)	4 (15.4%)	

Note: ICC, intrahepatic cholangiocarcinoma; ECC, extrahepatic cholangio-carcinoma; GBC, gallbladder carcinoma; BiIIN, biliary intraepithelial neoplasia Fisher's exact test for categorical data, Kruskal-Wallis test for continuous variables. # Chi-squared test with Monte Carlo simulation (2000 replicates).

Biliary intraepithelial neoplasia (BilIN) was present in 49/153 cases (32.0%), and its prevalence was significantly higher in ECC and GBC compared to ICC (ICC=15.7%; ECC=42.1%; GBC=53.8%; *p*=0.0002). Vascular and perineural invasion were present in 107 (69.9%) and 93 (60.8%) cases, respectively. Perineural invasion showed a significantly lower prevalence in ICC than ECC and GBC (ICC=44.3%; ECC=77.2%; GBC=69.2%; *p*=0.0005). Hepatitis virus infection (HBV and/or HCV) and cirrhosis prevalence were significantly higher in ICC patients (*p*=0.041 and *p*=0.005, respectively).

The pathologic stage of the 153 neoplasms was Stage I in 20, II in 51, III in 43, and IV in 39. ICC presented with more advanced stages at surgery compared to ECC and GBC (Table 1; *p*=0.034).

### Next-generation sequencing of 56 genes dissects cholangiocarcinoma molecular heterogeneity

DNA from all samples was successfully amplified in multiplex PCR for the 56 genes and an adequate library for deep sequencing was obtained. The mean read length was 78 base pairs and a mean coverage of 1800x was achieved, with 87.1% target bases covered more than 100x. A minimum coverage of 20x was obtained in all cases.

**Figure 1:**
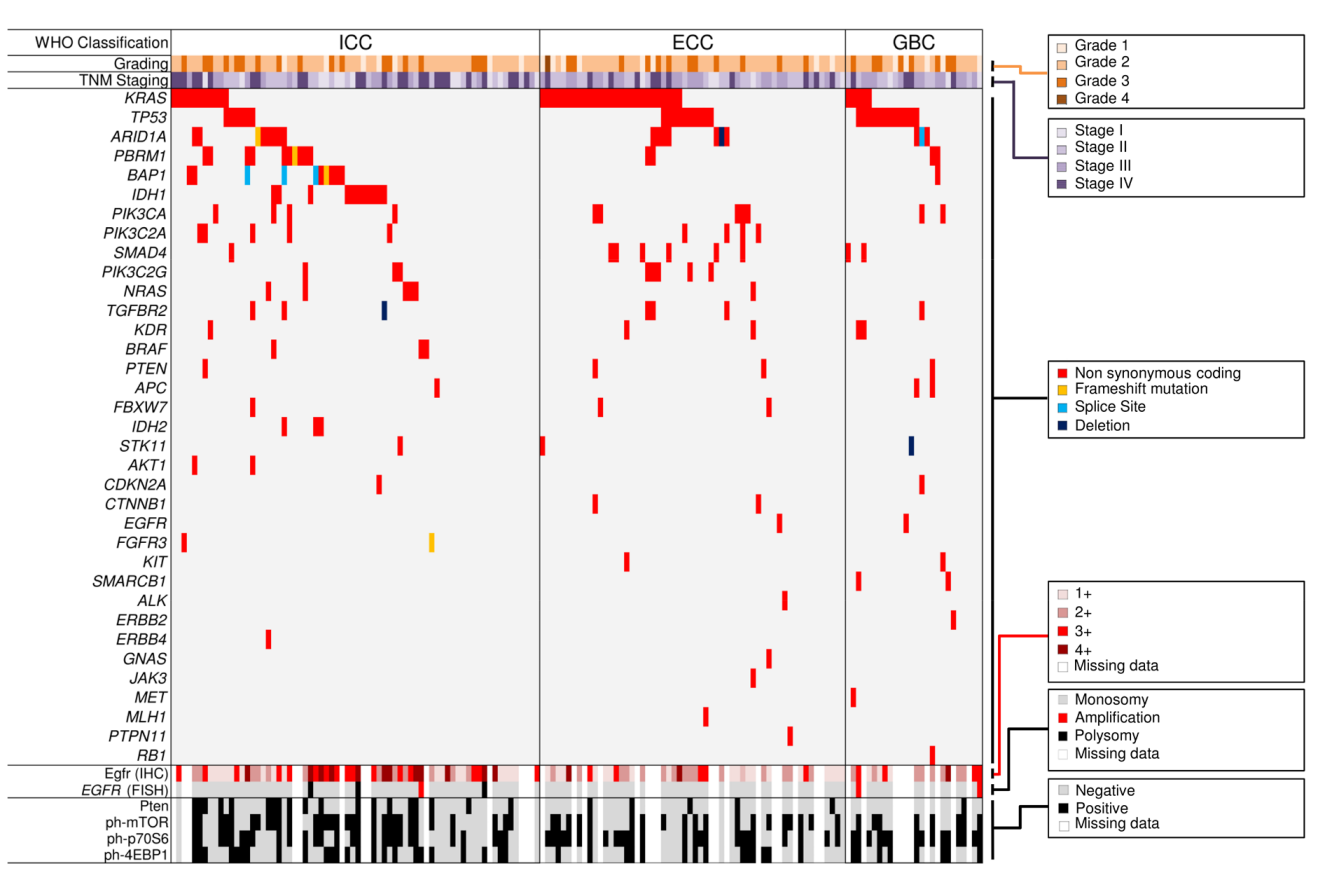
Mutation and immunohistochemical landscape of 153 primary biliary carcinomas The series includes 70 intrahepatic cholangiocarcinomas (ICC), 57 extrahepatic cholangiocarcinomas (ECC), and 26 gallbladder carcinomas (GBC). Significantly mutated genes are listed vertically in decreasing order of prevalence of nonsilent mutation. Colored rectangles indicate mutation category observed in a given gene and tumour. Tumour classifications and molecular features are as indicated in the boxes on the right. Immunoistochemistry phenotypes and FISH analysis results are shown in the bottom tracks. White boxes indicate unknown status or missing data.

**Figure 2:**
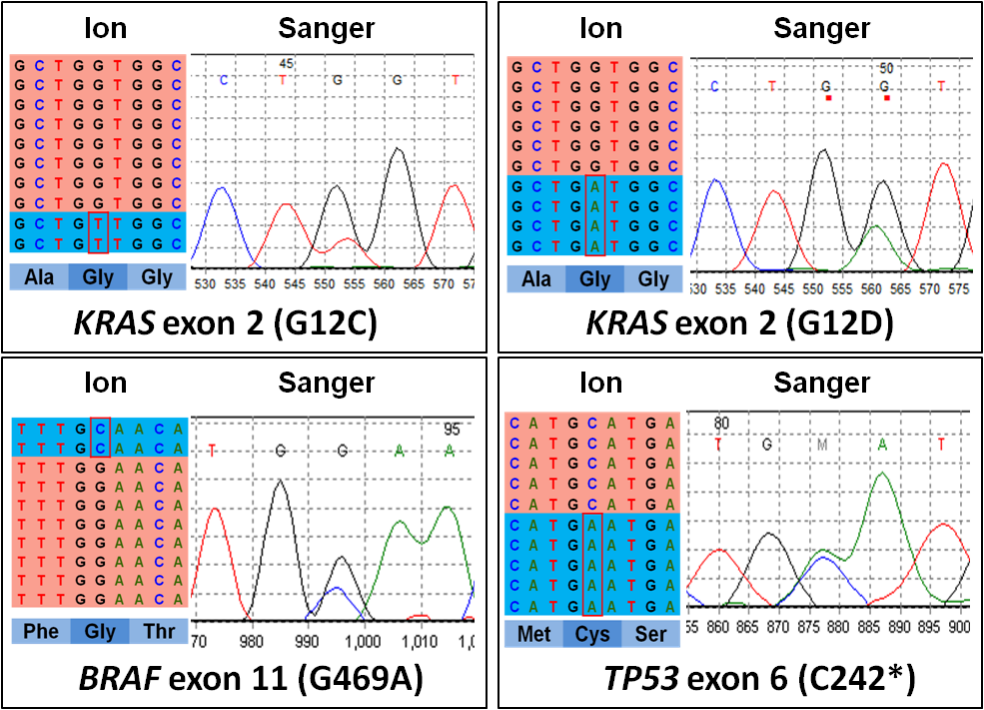
Representative examples of validation by Sanger sequencing of mutations identified using next generation sequencing On the left of each sample is the representation of the results of next-generation sequencing where the reads are aligned to the reference genome as provided by the Integrative Genomics Viewer (IGV v.2.1, Broad Institute) software. On the right is the representation of the results of Sanger sequencing.

At least one mutation was observed in 118/153 (77.1%) samples (Table 2, Figure 1); 60 cases (39.2%) showed concurrent mutations in different genes; 35 (22.9%) tumours showed no alterations in the 56 genes assayed. The most commonly mutated genes in the whole series were *KRAS* (28.1%), *TP53* (18.3%), *ARID1A* (11.8%), *IDH1/IDH2* (9.2%), *PBRM1* (9.2%), *BAP1* (7.2%), and *PIK3CA* (7.2%). Mutations in *BRAF*, *KRAS*, and *TP53* were all confirmed at Sanger sequencing (Figure 2).

**Table 2:** Mutational status of 153 biliary tract carcinomas

Gene	Total	Type of mutation					ICC (n= 70)	ECC (n= 57)	GBC (n= 26)	P-value*
		M	N	Fs	D	S				
AKT1	2	2					2			-
ALK	1(0.7%)	1						1(1.7%)		-
APC	3(2.0%)	2	1				1(1.4%)		2(7.7%)	-
ARID1A	18(11.8%)	10	5	1	1	1	8(11.4%)	7(12.3%)	3(11.5%)	0.999
BAP1	11(7.2%)	7		1		3	10(14.3%)		1(3.8%)	0.0097
BRAF	3(1.9%)	3					3(4.3%)			-
CDKN2A	2(1.3%)	1	1				1(1.4%)		1(3.8%)	-
CTNNB1	2(1.3%)	1	1					2(3.5%)		-
EGFR	2(1.3%)	2						1(1.7%)	1(3.8%)	-
ERBB2	1(0.7%)	1							1(3.8%)	-
ERBB4	1(0.7%)	1					1(1.4%)			-
FBXW7	3(2.0%)	3					1(1.4%)	2(3.5%)		-
FGFR3	2(1.3%)	1		1			2(2.8%)			-
GNAS	1(0.7%)	1						1(1.7%)		-
IDH1	11(7.2%)	11					11(15.7%)			0.0021
IDH2	3(2.0%)	3					3(4.3%)			-
JAK3	1(0.7%)	1						1(1.7%)		-
KDR	5(3.3%)	3	2				1(1.4%)	2(3.5%)	2(7.7%)	-
KIT	2(1.3%)	2						1(3.5%)	1(3.8%)	-
KRAS	43(28.1%)	43					11(15.7%)	7(47.4%)	5(19.2%)	0.0019
MET	1(0.7%)	1							1(3.8%)	-
MLH1	1(0.7%)	1						1(1.7%)		-
NRAS	6(3.9%)	6					5(9.3%)	1(1.7%)		0.421
PBRM1	14(9.2%)	8	5	1			10(14.3%)	2(3.5%)	2(7.7%)	0.182
PIK3CA	11(7.2%)	11					4(5.7%)	5(8.7%)	2(7.7%)	0.914
PIK3C2A	9(5.9%)	9					5(7.1%)	4(7.0%)		0.620
PIK3C2G	8(5.2%)	7	1				3(4.3%)	5(8.7%)		0.498
PTEN	4(2.6%)	4					1(1.4%)	2(3.5%)	1(3.8%)	-
PTPN11	1(0.7%)	1						1(1.7%)	0(0.0%)	-
RB1	1(0.7%)	1							1(3.8%)	-
RET	10.7%)	1						1(1.7%)		-
SMAD4	9(5.9%)	7	2				1(1.4%)	6(10.5%)	2(7.7%)	0.179
SMARCB1	2(1.3%)	2						0(0.0%)	2(7.7%)	-
STK11	3(2.0%)		2		1		1(1.4%)	1(2.2%)	1(3.8%)	-
TGFBR2	7(4.6%)	6			1		3(4.3%)	3(5.3%)	1(3.8%)	0.999
TP53	28(18.3%)	24	4				6(8.6%)	10(17.5%)	12(46.2%)	0.0019

Note: ICC, intrahepatic cholangiocarcinoma; ECC, extrahepatic cholangiocarcinoma; GBC, gallbladder carcinoma; M, missense mutation; N, nonsense mutation; F, frameshift mutation; D, deletion; S, splice site alteration.

* Fisher's exact test corrected for multiple comparisons was calculated If ≥ 6 mutated cases were observed.

Mutations were differently distributed across the different tumour subtypes: *IDH1*/*IDH2* (*p*=0.0005) were restricted to ICC and *BAP1* mutations were all found in ICC (*p*=0.0097) with the exception of one GBC, while *KRAS* (*p*=0.0019) and *TP53* (*p*=0.0019) were more represented in ECC and GBC, respectively (Table 2).

**Figure 3:**
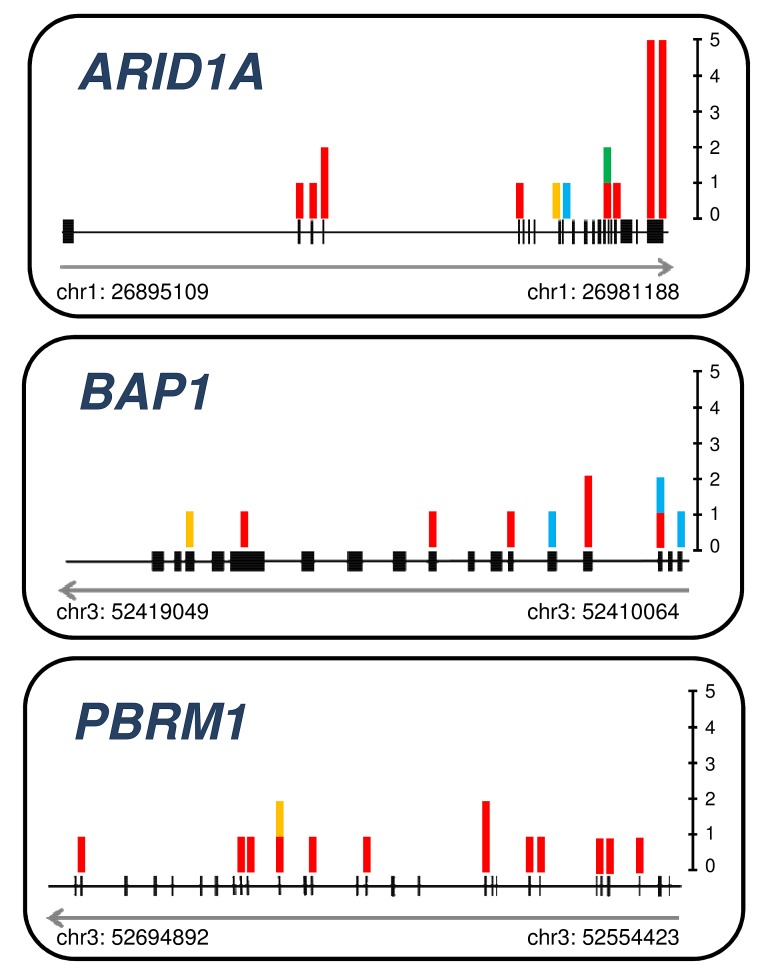
Somatic mutations detected in chromatin remodeling genes ARID1A, BAP1, and PBRM1 Schematic representation of *ARID1A*, *BAP1*, and *PBRM1* genes with the indication of the site of the somatic mutations identified in our study. Genomic coordinates are shown at the bottom track for each gene. Gray arrow indicates gene transcriptional direction. In black are represented the exons for each gene. Vertically, in correspondence of genomic location, bar chart indicate the type and number of mutations. Bar chart color is specific for mutation type: red, non synonymous coding; green, deletion; blue, splice site; yellow, frameshift.

ICC were characterized by a high prevalence of *IDH1/IDH2* mutations (20.0%) and the significant involvement of chromatin remodeling genes *PBRM1* (14.3%), *BAP1* (14.3%) and *ARID1A* (11.4%) (Figure 3), as described[32, 33]. *BAP1* and *IDH1* were mutually exclusive, whereas mutations in *IDH2* were always associated to *BAP1* mutations (3/3 cases). Eleven (15.7%) ICC had mutations in at least one of mTOR pathway genes: *AKT1* (2.8%), *PIK3CA* (5.7%), *PIK3C2A* (7.1%), *PIK3C2G* (4.3%), and *PTEN* (1.4%). Mutations in tyrosine kinase receptors were uncommon, with the exception of *TGFRB2* (4.3%). Of interest, most *NRAS* (5 of 6) and all *BRAF* (3 of 3) mutations clustered in ICC tumour subtype and were mutually exclusive with *KRAS* (15.7%). *TP53* was mutated in 6 cases (8.6%). Low prevalence mutations were found in *APC*, *CDKN2A*, *ERBB4*, *FBXW7, FGFR3*, *KDR/VEGFR2*, *SMAD4*, and *STK11*.

In ECC, *KRAS* was the most commonly mutated gene (47.4%), with codons 12, 13, 61 and 146 affected; one mutation was observed in *NRAS*, and none in *BRAF*. *TP53* was the second most mutated gene (17.5%). Excluding *ARID1A* (12.3%), chromatin-remodeling genes were occasionally involved (*PBRM1*: 3.5%), whereas 24.6% of ECCs showed mTOR pathway gene mutations, including *PIK3CA* (8.7%), *PIK3C2A* (7.0%), *PIK3C2G* (8.7%), and *PTEN* (3.5%). *SMAD4* mutations were observed in 6 cases (10.5%) and were mutually exclusive to *TGFBR2* mutations that were found in 3 cases (5.3%). Low prevalence mutations were found in *CTNNB1*, *FBXW7*, and *KDR/VEGFR2*. The *EGFR* T790M mutation was observed in one case [34].

GBC showed a high prevalence of *TP53* mutations (12/26, 46.2%), and in 6 cases *TP53* mutation was the only alteration detected. *KRAS* was mutated in 19.2% of cases. Chromatin remodeling genes were mutated in 30.8% of cases: *ARID1A*, 11.5%; *BAP1*, 3.8%; *PBRM1*, 7.7%; *SMARCB1*, 7.7%. MTOR pathway genes were mutated in 11.5% of cases: *PIK3CA* (7.7%) and *PTEN* (3.8%).

### mTOR pathway is dysregulated in all cholangiocarcinoma subtypes and Egfr is significantly overexpressed in intrahepatic cholangiocarcinomas

The results of immunohistochemistry are summarized in Table 3. We investigated mTOR pathway and Egfr expression in 113 neoplastic and 18 control cases. *EGFR* gene copy number was analyzed by FISH.

**Table 3:** EGFR immunohistochemical and gene copy number status, and mTOR pathway immunohistochemical profiling

Gene			Total	ICC	ECC	GBC	*P*-value*
Immunohistochemistry	EGFR	0	37 (32.7%)	17 (29.8%)	17 (42.5%)	3 (18.8%)	0.025
		1	36 (31.9%)	12 (21.0%)	16 (40.0%)	8 (50.0%)	
		2	25 (22.1%)	15 (26.4%)	6 (15.0%)	4 (25.0%)	
		3	15 (13.3%)	13 (22.8%)	1 (2.5%)	1 (6.2%)	
	PTEN	0	88 (77.9%)	39 (68.4%)	34 (85.0%)	15 (93.8%)	0.085
		1	25 (22.1%)	18 (31.6%)	6 (15.0%)	1 (6.2%)	
	ph-mTOR	0	55 (48.7%)	26 (45.6%)	21 (52.5%)	8 (50.0%)	0.785
		1	58 (51.3%)	31 (54.4%)	19 (47.5%)	8 (50.0%)	
	ph-p70S6	0	45 (39.8%)	29 (50.8%)	15 (37.5%)	1 (6.3%)	0.015
		1	68 (60.2%)	28 (49.2%)	25 (62.5%)	15 (93.7%)	
	ph-4EBP1	0	67 (59.3%)	34 (59.6%)	25 (62.5%)	8 (50.0%)	0.785
		1	46 (40.7%)	23 (40.4%)	15 (37.5%)	8 (50.0%)	
FISH	EGFR amplification		6 (5.3%)	4 (7.0%)	0 (0.0%)	2 (12.5%)	0.900

Note: ICC, intrahepatic cholangiocarcinoma; ECC, extrahepatic cholangiocarcinoma; GBC, gallbladder carcinoma.

* Fisher's exact test corrected for multiple comparisons.

A significant over-expression of the activated forms of mTOR and its effectors p70S6K and 4EBP1 was seen in most cancers with no significant differences among subtypes, but for p70S6K (Table 3). Of interest, the expression of phosphorylated ph-mTOR was significantly associated to the expression of the activated downstream effectors ph-4EBP1 and ph-p70S6K (*p*=0.05 and *p*=0.00012, respectively). Pten was significantly down-regulated in the whole series, particularly in ECC of common bile duct and in GBC: a weak cytoplasmic/nuclear immunolabeling was observed in most cases.

**Figure 4:**
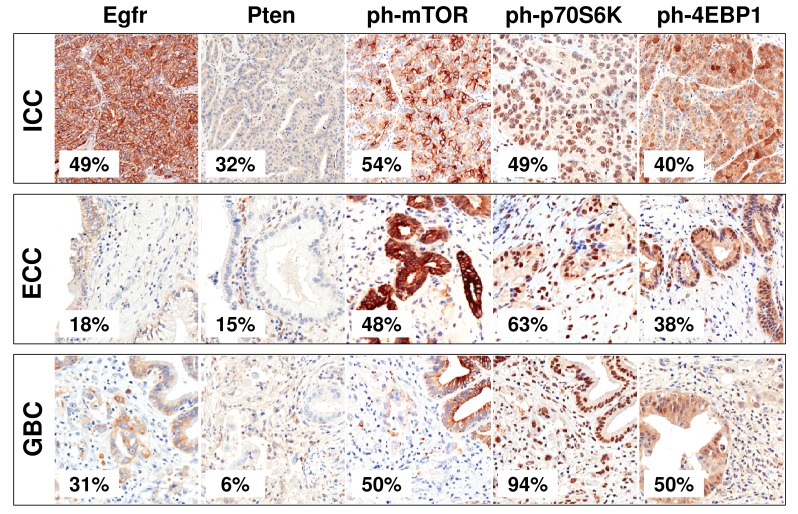
Immunohistochemical profiles of Egfr and mTOR pathway in cholangiocarcinomas Representative examples of immunohistochemical staining in cholangiocarcinoma samples. The prevalence of positive cases within the different tumour types is shown. Original magnfications 20x.

Egfr expression was significantly altered in biliary tract tumours, with different subtype specific profiles. Forty cancers (35.4%) labeled for Egfr with sharp membranous pattern and signals ranging from very strong to moderate (Table 3, Figure 1 and Figure 4). Strong (3+) overexpression was observed only in the ICC subtype. At FISH analysis, 3/113 (2.7%) tumours showed *EGFR* amplification (Figure 1).

There was no significant association between both EGFR and mTOR pathway immunophenotype and mutational status.

### TP53 mutation is an independent prognostic factor in cholangiocarcinoma

Survival data were available in 125 cases (ICC=51; ECC=50; GBC=24). Median survival was 31 months and 79 (63.2%) subjects were followed to their deaths from disease.

**Figure 5:**
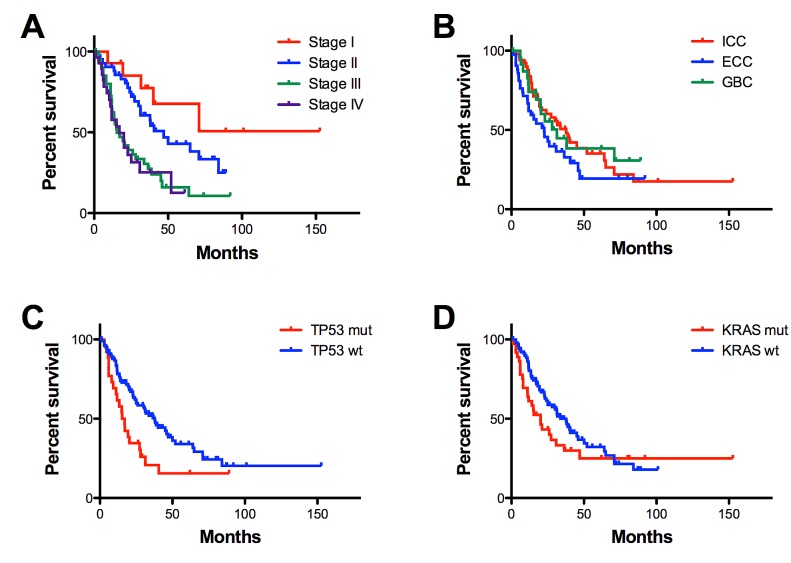
Overall survival according to pathological and mutational features Overall survival of 125 cholangiocarcinomas is significantly affected by tumour stage (*p*=0.0001) (A), tumour location (*p*=0.0176) (B), *TP53* (*p*=0.0043) (C) and *KRAS* (*p*=0.0162) (D) mutational status. Vertical axis indicates percent survival; horizontal axis shows time expressed in months. Kaplan–Meier and log-rank statistics were used to determine levels of significance.

At univariate analysis, the most significant predictors of cancer outcome were tumour stage (*p*=0.0001), *TP53* (*p*=0.0043) and *KRAS* (*p*=0.0162) mutations (Figure 5). Considering together *KRAS/BRAF* alterations, tumors characterized by mutations in *KRAS/BRAF* genes were associated to a worse patients' prognosis (*p*=0.0054). ICC showed a better outcome than ECC (*p*=0.018). No correlation emerged for any of the other clinicopathologic variables considered: sex, age, grade, vascular/perineural invasion.

Cox multivariate analysis including tumour subtype, stage, grade, vascular and perineural invasion, IDH1/2 mutations, *KRAS* and *TP53* mutations, identified only Stage III (*p*=0.005; OR 4.27; 95%C.I. 1.54-11.8), Stage IV (*p*=0.003; OR 4.85; 95%C.I. 1.68-14.0), and *TP53* mutations (*p*=0.002; OR 2.26; 95%C.I. 1.35-3.78) as being significantly associated with cancer-related death (Table 4).

**Table 4:** Multivariate survival analysis of 125 cholangiocarcinomas; median survival was 31 months and 79 subjects died of disease

Variable	Odds-ratio	95% C.I.	P-value
Stage = I	1	-	-
Stage = II	1.57	0.58-4.25	0.371
Stage = III	4.27	1.54-11.8	0.005
Stage = IV	4.85	1.68-14.0	0.003
Vascular invasion = yes	1.64	0.93-2.88	0.087
Perineural invasion = yes	0.63	0.36-1.08	0.093
TP53 = mutated	2.26	1.35-3.78	0.002
KRAS = mutated	1.51	0.91-2.51	0.110
Excluded variables			
Grade = 1	1	-	-
Grade = 2	1.38	0.57-3.37	0.474
Grade = 3	1.49	0.58-3.82	0.404
Class = ICC	1	-	-
Class = ECC	1.31	0.70-2.41	0.390
Class = GBC	0.67	0.30-1.49	0.323
IDH1/2 = mutated	1.26	0.50-3.17	0.631

Note: ICC, intrahepatic cholangiocarcinoma; ECC, extrahepatic cholangiocarcinoma; GBC, gallbladder carcinoma.

## DISCUSSION

The results of our next-generation mutational survey of 56 cancer genes in 153 biliary tree carcinomas can be summarized as follows: i) the vast majority (77.1%) of cancers harbours a driver-gene mutation; ii) the diverse sites of origin in the biliary tree have significantly different molecular profiles, and each site of origin also shows molecular heterogeneity; iii) targetable pathway alterations are present in 68% (104/153) of cancers, defining molecular cancer subclasses; iv) specific alterations are eligible for the investigational development of prognostic and non-invasive follow-up markers.

The vast majority of cancers (118/153, 77.1%) harboured at least one driver-gene mutation, and 39.2% (60/153 cases) showed concurrent mutations in two or more genes. *KRAS* was the most frequently mutated gene (28.1%), followed by *TP53* (18.3%), as reported in prior studies [9, 14, 35-38]. The recently described frequent involvement of the chromatin remodeling genes *ARID1A*, *PBRM1* and *BAP1* [9, 13] was also confirmed in our series, being found in 11.8%, 9.2% and 7.2% of cases, respectively. Univariate survival analysis showed that *KRAS* mutations were associated to a worse patients' prognosis confirming the univariate analysis previously described by Andersen [42]. Multivariate survival analysis identified tumour stage and *TP53* gene mutations as independent predictors of poor survival.

The different sites of origin showed significantly diverse molecular characteristics. *IDH1*/*IDH2* mutations were restricted to ICC (*p*=0.0005) and, with the exception of one GBC, *BAP1* mutations were all found in ICC (*p*=0.0097). ECC and GBC were characterized by a high prevalence of *KRAS* (*p*=0.0019) and *TP53* (*p*=0.0019) mutations, respectively.

The standard of care of biliary tract cancer is based on the combination of cisplatin and gemcitabine [39]. To date, clinical trials with targeted therapies for advanced biliary tract cancers have failed to produce significant benefits [11], and ongoing studies are exploring the combination of chemotherapy with novel MAPK/ERK Kinase (MEK) and mTOR inhibitors [40]. However, neither previous nor ongoing studies have considered evaluating tumour response against genetic alterations. Our identification of molecular subclasses with specific drug actionable pathway alterations in 104/153 (68.0%) tumours may tailor the design of trials based on the molecular selection of patients, irrespective of the site of origin, where actionable signaling pathways include tyrosine-kinase receptors (TKR), RAS/RAF/MAPK/ERK, mTOR, and TGF-ß.

Mutations in tyrosine kinase receptors (*ALK*, *EGFR*, *ERBB2*, *ERBB4*, *FGFR3*, *MET*, *KIT*, *KDR/VEGFR2*) potentially amenable to target therapies were found in 9.2% of cases, with a higher prevalence in GBC (6/26 cases, 23.1%) than in ICC (4/70 cases, 5.7%) and ECC (4/57, 7.0%). In spite of a relatively high prevalence of Egfr overexpression detected by immunohistochemistry (35.4% of cases with 2+ or 3+), only two cases had *EGFR* mutations and three had gene amplification, confirming the low prevalence reported in the literature in cholangiocarcinomas unassociated with chronic liver disease [14, 38, 41-44].

Mutations in components of the *RAS* pathway (*KRAS*, *NRAS*, *BRAF*) were observed in 34% of the whole series. In particular *KRAS* was the most frequently mutated gene in the 153 tumours (28.1%). *KRA*S mutations have been described in most of prior studies [9, 14, 35-38]. *KRAS*/*NRAS*/*BRAF* mutations were mutually exclusive, and the highest mutation prevalence in *RAS* pathway was observed in ECC (49.1%) *vs.* ICC (27.1%) and GBC (19.2%). Of note, *RAS* mutations sensitize tumours to MEK inhibitors, highlighting the importance of these mutations in the use of targeted therapies [11, 40].

MTOR pathway relevance in biliary tract cancers is suggested by our immunohistochemical detection of activated forms of mTor and its downstream effectors in 51.3% of the cancers. The molecular basis of this activation in a proportion of cases is the mutation in one of the genes involved in this pathway (i.e., *AKT*, *FBXW7*, *PIK3CA*, *PIK3C2A*, *PIK3C2G*, *PTEN*). This suggests that mTOR inhibitors might play a role in this molecular subgroup of patients.

TGF-ß/Smad signaling was altered in 16/153 cases (10.5%), 9 of which were ECC (9/57, 15.8%). Our finding supports previous studies demonstrating the involvement of this pathway in cholangiocarcinomas[45, 46]. Our study may have underestimated the involvement of this pathway, as only the mutational status of *TGFBR2* and *SMAD4* genes was investigated, and the latter is frequently inactivated by mechanisms different from intragenic mutations, such as homozygous deletions, which would not be detected by the techniques used in this study.

Alterations in chromatin remodeling genes (*ARID1A*, *BAP1*, *IDH1*, *IDH2*, *PBRM1*, *SMARCB1*) were found in 30.7% (47/153) of cancers in our series, confirming recent reports on the significant involvement of these genes in cholangiocarcinoma [9, 13]. Mutations in these genes appear to be either specific to ICC, as is the case of *IDH1* and *IDH2* (*p*=0.0005), or cluster within this cancer type as is the case for *ARID1A*, *BAP1*, and *PBRM1* that were found in 34.3% (24/70) of ICC. The open challenge is now to translate knowledge of the targeting of these genes to improved patient care through either the development of new disease specific markers or of therapy targets. Of interest, *IDH1/2* mutated cancers accumulate 2-hydroxyglutarate in tumour tissue and release the molecule in blood, and the measurement of 2-hydroxyglutarate might be used as both a surrogate biomarker for *IDH1/2* mutational status and a non-invasive test for the assessment of tumour burden in ICC [47].

Our study is limited by the number of genes analyzed; 35 cancers (22.9%) showed no alterations in the 56 genes assayed, including 50 genes from a commercial panel and a custom panel exploring 6 recently discovered cholangiocarcinoma genes [9, 13]. Mechanisms different from intragenic mutations, such as amplifications, deletions, translocations, and epigenetic anomalies should also be addressed.

In conclusion, we demonstrate that specific molecular alterations are associated to different cholangiocarcinomas categories and that potentially drug actionable pathways are evident in 68% of cases. These data further support the pathological and molecular heterogeneity characterizing biliary tree carcinomas. In currently designed clinical trials, cholangiocarcinomas are grouped together irrespective of their intrahepatic or extrahepatic site of origin. Our study shows that ICC and ECC should be considered separately, since they have different molecular characteristics. However, in the advent of molecular designed clinical trials, it would be appropriate to consider them together but only for the proportion of ICC and ECC sharing common molecular alterations.

We also show that a high-throughput next-generation sequencing analysis can be successfully applied using low amounts of DNA from routinely processed paraffin tissues. Such a time- and cost-effective analysis is the basis to significantly improve the development of personalized treatments for cholangiocarcinoma patients, and their early access to innovative drugs.

## MATERIALS AND METHODS

### Cases

A retrospective series (1990-2011) of 153 surgically-resected primary biliary cancers were retrieved from the FFPE archives of the ARC-Net Biobank at Verona University Hospital under the local ethics committee approval (n. prog. 1959). All cases were re-classified according to WHO 2010 [1], and included 70 ICC, 57 ECC and 26 GBC. Staging was according to AJCC/UICC 7th edition[26]. Matched normal liver was used to determine the somatic/germline nature of mutations.

In 113 cases (57 ICC, 40 ECC, 16 GBC), sufficient material for the construction of 1-mm cores tissue microarrays (TMAs) was available. Eighteen non-neoplastic controls (8 normal biliary duct and 10 chronic cholecystitis) were included in the TMAs. Three tissue cores per case were analyzed.

### DNA extraction and qualification

DNA was prepared after enrichment for neoplastic cellularity to at least 70% using manual microdissection of 10 consecutive 4-μm FFPE sections, purified using the QIAamp DNA FFPE Tissue Kit (Qiagen), and qualified as reported elsewhere [24, 27].

### Next-Generation Sequencing of Multiplex PCR Amplicons

Two multigene panels were used: the 50-gene Ion AmpliSeq Cancer Hotspot panel v2 (Life Technologies) and an AmpliSeq custom panel targeting 6 genes not included in the commercial panel. The first explores selected regions of 50 cancer- genes: ABL1, AKT1, ALK, APC, ATM, BRAF, CDH1, CDKN2A, CSF1R, CTNNB1, EGFR, ERBB2, ERBB4, EZH2, FBXW7, FGFR1, FGFR2, FGFR3, FLT3, GNA11, GNAS, GNAQ, HNF1A, HRAS, IDH1, IDH2, JAK2, JAK3, KDR/VEGFR2, KIT, KRAS, MET, MLH1, MPL, NOTCH1, NPM1, NRAS, PDGFRA, PIK3CA, PTEN, PTPN11, RB1, RET, SMAD4, SMARCB1, SMO, SRC, STK11, TP53, VHL. Details on target regions of the commercial panel are at http://www.lifetechnologies.com. The custom panel targets 6 genes selected upon the results of published ICC exome sequencing: ARID1A, BAP1, PBRM1, PIK3C2A, PIK3C2G, TGFBR2[13]. Details of the custom panel are in Supplementary Table 1.

Twenty nanograms of DNA were used for each multiplex PCR amplification. Emulsion PCR was performed with the OneTouch2 system (Life Technologies). The quality of the obtained libraries was evaluated by the Agilent 2100 Bioanalyzer on-chip electrophoresis (Agilent Technologies). Sequencing was run on the Ion Torrent Personal Genome Machine (PGM, Life Technologies) loaded with 316 (50-gene panel) or 318 chips (6-gene panel). Data analysis, including alignment to the hg19 human reference genome and variant calling, was done using the Torrent Suite Software v.3.6 (Life Technologies). Filtered variants were annotated using the SnpEff software v.3.1. Alignments were visually verified with the Integrative Genomics Viewer; IGV v.2.2, Broad Institute.

### DNA Sanger Sequencing

To validate the mutations detected by deep sequencing, BRAF (exon 11), KRAS (exon 2), and TP53 (exons 2, 5, 6, 7, 8) were analyzed by Sanger sequencing [28].

### Immunohistochemistry

The immunohistochemical expression of Egfr (Dako), Pten (Abnova Corporation), and of the phosphorylated forms of mTOR (Ser2448, clone 49F9; Cell Signaling) and its downstream effectors 4EBP1 (Thr37/46, clone 236B4; Cell Signaling) and p70S6K (Thr389, clone 1A5; Cell Signaling) was examined on consecutive 4-μm FFPE TMA sections. Appropriate positive and negative controls were run concurrently.

Egfr expression was scored according to the EGFR pharmDx protocol (Dako): 0, no staining or membrane staining in ≤10% cancer cells; 1+, faint and partial membrane staining in >10% cancer cells; 2+, moderate and complete membrane staining in >10% cancer cells; 3+, strong and complete membrane staining in >10% cancer cells. Cases were then classified in two groups Egfr-positive (2+ and 3+) or Egfr-negative (0 and 1+).

Pten expression was considered positive if more than 50% of neoplastic cells showed a moderate/strong nuclear and cytoplasmic immunoreaction[29].

Staining for phosphorylated markers (ph-mTOR, ph-4EBP1, and ph-p70S6K) was considered positive when tumour cells showed cytoplasmic and/or nuclear staining with equal to stronger intensity compared with that of endothelial cells [30].

### Fluorescent in situ hybridization (FISH)

The EGFR gene copy number status was assessed applying the Vysis EGFR/CEP7 Probe Kit (Vysis/Abbott Molecular). At least 50 representative nuclei per specimen were scored and EGFR-amplification was defined as described [31].

### Statistical analysis

Kruskal-Wallis test, Chi-squared test with Monte Carlo simulation, and Fisher's exact test corrected for multiple comparisons were used as appropriate. For comparison of Kaplan-Meier survival curves, Mantel-Cox log-rank test was used; for multivariate survival analysis, stepwise Cox proportional hazards regression was used; selection of the best model was performed using the “backward elimination” algorithm. For all the analyses a p-value below 0.05 was considered significant. Graphs and univariate analyses were performed using GraphPad Prism® version 5.00 for Mac (GraphPad Software, San Diego California USA), multivariate Cox regression was done with R v. 3.0.2, using survival library v.2.37-4.

## SUPPLEMENTARY TABLE



## Abbreviations

ECC: extrahepatic cholangiocarcinomas
FFPE: formalin-fixed paraffin-embedded
GBC: gallbladder carcinomas
ICC: intrahepatic cholangiocarcinomas
NGS: next-generation sequencing
