# Supplementary material for: Multigene mutational profiling of cholangiocarcinomas identifies actionable molecular subgroups
Source: Oncotarget. 2014 May 1;5(9):2839–52. doi: 10.18632/oncotarget.1943 (PMC4058049; doi:10.18632/oncotarget.1943)
Supplement: Supplementary file 1 [file oncotarget-05-2839-s001.pdf]

Multigene mutational profiling of cholangiocarcinomas identifies actionable molecular subgroups

Supplementary Material

Supplementary Table 1: Targeted regions of the Ampliseq custom panel exploring 6 genes: *ARID1A*, *BAP1*, *PBRM1*, *PIK3C2A*, *PIK3C2G*, *TGFBR2.*

Chromosome	Start amplicon	End amplicon	Gene
chr1	27023113	27023236	ARID1A
chr1	27023236	27023335	ARID1A
chr1	27023309	27023418	ARID1A
chr1	27023415	27023518	ARID1A
chr1	27023502	27023595	ARID1A
chr1	27023684	27023776	ARID1A
chr1	27023697	27023836	ARID1A
chr1	27024034	27024137	ARID1A
chr1	27056019	27056119	ARID1A
chr1	27056119	27056237	ARID1A
chr1	27056232	27056358	ARID1A
chr1	27056358	27056438	ARID1A
chr1	27057577	27057702	ARID1A
chr1	27057702	27057827	ARID1A
chr1	27057827	27057961	ARID1A
chr1	27057956	27058065	ARID1A
chr1	27058060	27058149	ARID1A
chr1	27059099	27059227	ARID1A
chr1	27059227	27059352	ARID1A
chr1	27087293	27087411	ARID1A
chr1	27087410	27087546	ARID1A
chr1	27087545	27087639	ARID1A
chr1	27087817	27087931	ARID1A
chr1	27087931	27088018	ARID1A
chr1	27088591	27088717	ARID1A
chr1	27088715	27088836	ARID1A
chr1	27088836	27088910	ARID1A
chr1	27089401	27089525	ARID1A
chr1	27089519	27089642	ARID1A
chr1	27089632	27089766	ARID1A
chr1	27089766	27089849	ARID1A
chr1	27092652	27092767	ARID1A
chr1	27092767	27092866	ARID1A
chr1	27092866	27092982	ARID1A
chr1	27093004	27093112	ARID1A
chr1	27094197	27094298	ARID1A

chr1	27094298	27094424	ARID1A
chr1	27094424	27094540	ARID1A
chr1	27097483	27097589	ARID1A
chr1	27097589	27097698	ARID1A
chr1	27097698	27097779	ARID1A
chr1	27097779	27097903	ARID1A
chr1	27098921	27099047	ARID1A
chr1	27099045	27099169	ARID1A
chr1	27099169	27099252	ARID1A
chr1	27099250	27099358	ARID1A
chr1	27099358	27099473	ARID1A
chr1	27099473	27099563	ARID1A
chr1	27099720	27099843	ARID1A
chr1	27099842	27099961	ARID1A
chr1	27099961	27100055	ARID1A
chr1	27100047	27100153	ARID1A
chr1	27100151	27100274	ARID1A
chr1	27100239	27100371	ARID1A
chr1	27100371	27100453	ARID1A
chr1	27100757	27100894	ARID1A
chr1	27100894	27101000	ARID1A
chr1	27101000	27101091	ARID1A
chr1	27101088	27101192	ARID1A
chr1	27101191	27101310	ARID1A
chr1	27101310	27101441	ARID1A
chr1	27101437	27101533	ARID1A
chr1	27101533	27101650	ARID1A
chr1	27101648	27101761	ARID1A
chr1	27101966	27102045	ARID1A
chr1	27102045	27102144	ARID1A
chr1	27102144	27102252	ARID1A
chr1	27105423	27105540	ARID1A
chr1	27105540	27105641	ARID1A
chr1	27105640	27105756	ARID1A
chr1	27105756	27105848	ARID1A
chr1	27105848	27105962	ARID1A
chr1	27105962	27106049	ARID1A
chr1	27106046	27106173	ARID1A
chr1	27106172	27106284	ARID1A
chr1	27106282	27106363	ARID1A
chr1	27106363	27106494	ARID1A
chr1	27106494	27106573	ARID1A
chr1	27106571	27106663	ARID1A
chr1	27106661	27106760	ARID1A
chr1	27106760	27106852	ARID1A

chr1	27106850	27106958	ARID1A
chr1	27106956	27107069	ARID1A
chr1	27107068	27107181	ARID1A
chr1	27107181	27107302	ARID1A
chr3	30648360	30648456	TGFBR2
chr3	30648446	30648539	TGFBR2
chr3	30664759	30664885	TGFBR2
chr3	30686137	30686253	TGFBR2
chr3	30686253	30686356	TGFBR2
chr3	30686356	30686464	TGFBR2
chr3	30691680	30691778	TGFBR2
chr3	30691778	30691901	TGFBR2
chr3	30691901	30692002	TGFBR2
chr3	30713069	30713153	TGFBR2
chr3	30713153	30713230	TGFBR2
chr3	30713230	30713339	TGFBR2
chr3	30713338	30713467	TGFBR2
chr3	30713466	30713577	TGFBR2
chr3	30713577	30713695	TGFBR2
chr3	30713695	30713788	TGFBR2
chr3	30713783	30713903	TGFBR2
chr3	30713903	30713983	TGFBR2
chr3	30715540	30715663	TGFBR2
chr3	30715663	30715788	TGFBR2
chr3	30729817	30729943	TGFBR2
chr3	30729943	30730069	TGFBR2
chr3	30732806	30732931	TGFBR2
chr3	30732931	30733059	TGFBR2
chr3	30733059	30733169	TGFBR2
chr3	52436209	52436322	BAP1
chr3	52436317	52436457	BAP1
chr3	52436456	52436590	BAP1
chr3	52436538	52436650	BAP1
chr3	52436650	52436768	BAP1
chr3	52436698	52436809	BAP1
chr3	52436809	52436940	BAP1
chr3	52436993	52437126	BAP1
chr3	52437126	52437232	BAP1
chr3	52437230	52437364	BAP1
chr3	52437369	52437493	BAP1
chr3	52437493	52437587	BAP1
chr3	52437585	52437667	BAP1
chr3	52437666	52437798	BAP1
chr3	52437798	52437922	BAP1
chr3	52437916	52438046	BAP1

chr3	52438403	52438530	BAP1
chr3	52438530	52438653	BAP1
chr3	52439064	52439187	BAP1
chr3	52439187	52439321	BAP1
chr3	52439320	52439428	BAP1
chr3	52439726	52439857	BAP1
chr3	52439857	52439978	BAP1
chr3	52440194	52440317	BAP1
chr3	52440313	52440446	BAP1
chr3	52440706	52440810	BAP1
chr3	52440809	52440937	BAP1
chr3	52440937	52441027	BAP1
chr3	52441114	52441202	BAP1
chr3	52441202	52441297	BAP1
chr3	52441295	52441411	BAP1
chr3	52441427	52441526	BAP1
chr3	52441889	52442000	BAP1
chr3	52442000	52442081	BAP1
chr3	52442081	52442170	BAP1
chr3	52442429	52442554	BAP1
chr3	52442552	52442673	BAP1
chr3	52443462	52443581	BAP1
chr3	52443581	52443710	BAP1
chr3	52443730	52443862	BAP1
chr3	52443751	52443884	BAP1
chr3	52582021	52582118	PBRM1
chr3	52582117	52582211	PBRM1
chr3	52582206	52582301	PBRM1
chr3	52584383	52584453	PBRM1
chr3	52584451	52584564	PBRM1
chr3	52584564	52584689	PBRM1
chr3	52584689	52584812	PBRM1
chr3	52584832	52584919	PBRM1
chr3	52588688	52588819	PBRM1
chr3	52588817	52588899	PBRM1
chr3	52588898	52588977	PBRM1
chr3	52595686	52595794	PBRM1
chr3	52595794	52595907	PBRM1
chr3	52595907	52596035	PBRM1
chr3	52597179	52597266	PBRM1
chr3	52597266	52597367	PBRM1
chr3	52597367	52597455	PBRM1
chr3	52597455	52597559	PBRM1
chr3	52598017	52598121	PBRM1
chr3	52598107	52598178	PBRM1

chr3	52610566	52610654	PBRM1
chr3	52610575	52610698	PBRM1
chr3	52613018	52613142	PBRM1
chr3	52613142	52613265	PBRM1
chr3	52620331	52620413	PBRM1
chr3	52620409	52620501	PBRM1
chr3	52620501	52620588	PBRM1
chr3	52620585	52620667	PBRM1
chr3	52620667	52620782	PBRM1
chr3	52621293	52621364	PBRM1
chr3	52621359	52621452	PBRM1
chr3	52621410	52621488	PBRM1
chr3	52623002	52623107	PBRM1
chr3	52623107	52623209	PBRM1
chr3	52623209	52623327	PBRM1
chr3	52637473	52637594	PBRM1
chr3	52637502	52637633	PBRM1
chr3	52637646	52637744	PBRM1
chr3	52637671	52637783	PBRM1
chr3	52643260	52643373	PBRM1
chr3	52643373	52643483	PBRM1
chr3	52643481	52643614	PBRM1
chr3	52643609	52643733	PBRM1
chr3	52643733	52643828	PBRM1
chr3	52643826	52643947	PBRM1
chr3	52643946	52644034	PBRM1
chr3	52649240	52649366	PBRM1
chr3	52649365	52649445	PBRM1
chr3	52649445	52649528	PBRM1
chr3	52651206	52651327	PBRM1
chr3	52651327	52651451	PBRM1
chr3	52651420	52651522	PBRM1
chr3	52651507	52651609	PBRM1
chr3	52661255	52661328	PBRM1
chr3	52661327	52661408	PBRM1
chr3	52661395	52661463	PBRM1
chr3	52662843	52662946	PBRM1
chr3	52662946	52663012	PBRM1
chr3	52668538	52668606	PBRM1
chr3	52668588	52668705	PBRM1
chr3	52668705	52668820	PBRM1
chr3	52668820	52668894	PBRM1
chr3	52675881	52676001	PBRM1
chr3	52676000	52676119	PBRM1
chr3	52677200	52677290	PBRM1

chr3	52677290	52677410	PBRM1
chr3	52678715	52678833	PBRM1
chr3	52678744	52678872	PBRM1
chr3	52682328	52682419	PBRM1
chr3	52682403	52682479	PBRM1
chr3	52685666	52685741	PBRM1
chr3	52685738	52685837	PBRM1
chr3	52685801	52685911	PBRM1
chr3	52692123	52692208	PBRM1
chr3	52692208	52692318	PBRM1
chr3	52692318	52692401	PBRM1
chr3	52696017	52696126	PBRM1
chr3	52696126	52696229	PBRM1
chr3	52696229	52696352	PBRM1
chr3	52702494	52702601	PBRM1
chr3	52702563	52702637	PBRM1
chr3	52712432	52712550	PBRM1
chr3	52712550	52712645	PBRM1
chr3	52712616	52712689	PBRM1
chr3	52713513	52713601	PBRM1
chr3	52713601	52713721	PBRM1
chr3	52713721	52713806	PBRM1
chr11	17111142	17111256	PIK3C2A
chr11	17111256	17111330	PIK3C2A
chr11	17111330	17111414	PIK3C2A
chr11	17111414	17111519	PIK3C2A
chr11	17112899	17112971	PIK3C2A
chr11	17112963	17113047	PIK3C2A
chr11	17113047	17113151	PIK3C2A
chr11	17113151	17113270	PIK3C2A
chr11	17113446	17113560	PIK3C2A
chr11	17113560	17113683	PIK3C2A
chr11	17113635	17113748	PIK3C2A
chr11	17115752	17115864	PIK3C2A
chr11	17115864	17115975	PIK3C2A
chr11	17118525	17118647	PIK3C2A
chr11	17118675	17118756	PIK3C2A
chr11	17118756	17118831	PIK3C2A
chr11	17118817	17118888	PIK3C2A
chr11	17121289	17121408	PIK3C2A
chr11	17121386	17121478	PIK3C2A
chr11	17121471	17121580	PIK3C2A
chr11	17122830	17122906	PIK3C2A
chr11	17122876	17122948	PIK3C2A
chr11	17122948	17123052	PIK3C2A

chr11	17124153	17124249	PIK3C2A
chr11	17124249	17124335	PIK3C2A
chr11	17124330	17124404	PIK3C2A
chr11	17126643	17126725	PIK3C2A
chr11	17126709	17126819	PIK3C2A
chr11	17126814	17126884	PIK3C2A
chr11	17131896	17132016	PIK3C2A
chr11	17132016	17132123	PIK3C2A
chr11	17135825	17135942	PIK3C2A
chr11	17135942	17136064	PIK3C2A
chr11	17138966	17139050	PIK3C2A
chr11	17139050	17139147	PIK3C2A
chr11	17139147	17139225	PIK3C2A
chr11	17140116	17140231	PIK3C2A
chr11	17140238	17140314	PIK3C2A
chr11	17140715	17140799	PIK3C2A
chr11	17140799	17140919	PIK3C2A
chr11	17141277	17141378	PIK3C2A
chr11	17141372	17141446	PIK3C2A
chr11	17141429	17141499	PIK3C2A
chr11	17143698	17143808	PIK3C2A
chr11	17143749	17143851	PIK3C2A
chr11	17144169	17144244	PIK3C2A
chr11	17144244	17144339	PIK3C2A
chr11	17144320	17144417	PIK3C2A
chr11	17150784	17150903	PIK3C2A
chr11	17150903	17150996	PIK3C2A
chr11	17153405	17153490	PIK3C2A
chr11	17153450	17153533	PIK3C2A
chr11	17156361	17156440	PIK3C2A
chr11	17156440	17156548	PIK3C2A
chr11	17156548	17156658	PIK3C2A
chr11	17156683	17156769	PIK3C2A
chr11	17157917	17158037	PIK3C2A
chr11	17158037	17158142	PIK3C2A
chr11	17158130	17158222	PIK3C2A
chr11	17167162	17167250	PIK3C2A
chr11	17167250	17167355	PIK3C2A
chr11	17167319	17167396	PIK3C2A
chr11	17167501	17167565	PIK3C2A
chr11	17168948	17169071	PIK3C2A
chr11	17169070	17169144	PIK3C2A
chr11	17169144	17169242	PIK3C2A
chr11	17170192	17170271	PIK3C2A
chr11	17170267	17170359	PIK3C2A

chr11	17171960	17172037	PIK3C2A
chr11	17172037	17172164	PIK3C2A
chr11	17172164	17172269	PIK3C2A
chr11	17176978	17177097	PIK3C2A
chr11	17177034	17177151	PIK3C2A
chr11	17177151	17177257	PIK3C2A
chr11	17190191	17190283	PIK3C2A
chr11	17190277	17190359	PIK3C2A
chr11	17190359	17190460	PIK3C2A
chr11	17190460	17190535	PIK3C2A
chr11	17190535	17190660	PIK3C2A
chr11	17190660	17190765	PIK3C2A
chr11	17190765	17190884	PIK3C2A
chr11	17190884	17190984	PIK3C2A
chr11	17190984	17191068	PIK3C2A
chr11	17191068	17191158	PIK3C2A
chr11	17191158	17191277	PIK3C2A
chr11	17191277	17191344	PIK3C2A
chr12	18434980	18435048	PIK3C2G
chr12	18435046	18435142	PIK3C2G
chr12	18435142	18435249	PIK3C2G
chr12	18435249	18435328	PIK3C2G
chr12	18435328	18435415	PIK3C2G
chr12	18435415	18435521	PIK3C2G
chr12	18435475	18435551	PIK3C2G
chr12	18435617	18435724	PIK3C2G
chr12	18435684	18435753	PIK3C2G
chr12	18439712	18439822	PIK3C2G
chr12	18439823	18439920	PIK3C2G
chr12	18443711	18443786	PIK3C2G
chr12	18443771	18443843	PIK3C2G
chr12	18443838	18443913	PIK3C2G
chr12	18443890	18443991	PIK3C2G
chr12	18466832	18466934	PIK3C2G
chr12	18466908	18467008	PIK3C2G
chr12	18467001	18467068	PIK3C2G
chr12	18473827	18473902	PIK3C2G
chr12	18473906	18473986	PIK3C2G
chr12	18477887	18478005	PIK3C2G
chr12	18491276	18491391	PIK3C2G
chr12	18491369	18491463	PIK3C2G
chr12	18496160	18496272	PIK3C2G
chr12	18496295	18496420	PIK3C2G
chr12	18499523	18499599	PIK3C2G
chr12	18499599	18499714	PIK3C2G

chr12	18499713	18499832	PIK3C2G
chr12	18524053	18524128	PIK3C2G
chr12	18524102	18524186	PIK3C2G
chr12	18524183	18524267	PIK3C2G
chr12	18524243	18524322	PIK3C2G
chr12	18534577	18534701	PIK3C2G
chr12	18534701	18534802	PIK3C2G
chr12	18534798	18534897	PIK3C2G
chr12	18544027	18544109	PIK3C2G
chr12	18544103	18544196	PIK3C2G
chr12	18544192	18544268	PIK3C2G
chr12	18552556	18552631	PIK3C2G
chr12	18552558	18552657	PIK3C2G
chr12	18552741	18552813	PIK3C2G
chr12	18573815	18573926	PIK3C2G
chr12	18573926	18574049	PIK3C2G
chr12	18576802	18576896	PIK3C2G
chr12	18576896	18577023	PIK3C2G
chr12	18641297	18641414	PIK3C2G
chr12	18641393	18641509	PIK3C2G
chr12	18641499	18641580	PIK3C2G
chr12	18641567	18641640	PIK3C2G
chr12	18644305	18644385	PIK3C2G
chr12	18644349	18644434	PIK3C2G
chr12	18644485	18644564	PIK3C2G
chr12	18648907	18649018	PIK3C2G
chr12	18649003	18649084	PIK3C2G
chr12	18649082	18649155	PIK3C2G
chr12	18650467	18650581	PIK3C2G
chr12	18650581	18650655	PIK3C2G
chr12	18650647	18650733	PIK3C2G
chr12	18656134	18656253	PIK3C2G
chr12	18656253	18656349	PIK3C2G
chr12	18656349	18656420	PIK3C2G
chr12	18658170	18658261	PIK3C2G
chr12	18658259	18658346	PIK3C2G
chr12	18658346	18658448	PIK3C2G
chr12	18691042	18691157	PIK3C2G
chr12	18691121	18691201	PIK3C2G
chr12	18699171	18699248	PIK3C2G
chr12	18699248	18699361	PIK3C2G
chr12	18699361	18699436	PIK3C2G
chr12	18715547	18715673	PIK3C2G
chr12	18715672	18715761	PIK3C2G
chr12	18715761	18715878	PIK3C2G

chr12	18716224	18716327	PIK3C2G
chr12	18716324	18716403	PIK3C2G
chr12	18716402	18716514	PIK3C2G
chr12	18719824	18719899	PIK3C2G
chr12	18747382	18747454	PIK3C2G
chr12	18747383	18747478	PIK3C2G
chr12	18762388	18762463	PIK3C2G
chr12	18762449	18762523	PIK3C2G
chr12	18762523	18762614	PIK3C2G
chr12	18793285	18793406	PIK3C2G
chr12	18793406	18793487	PIK3C2G
chr12	18793487	18793567	PIK3C2G
chr12	18800706	18800827	PIK3C2G
chr12	18800828	18800901	PIK3C2G
chr12	18800910	18801028	PIK3C2G
